# A radiomics nomogram for predicting cytokeratin 19–positive hepatocellular carcinoma: a two-center study

**DOI:** 10.3389/fonc.2023.1174069

**Published:** 2023-04-27

**Authors:** Liqing Zhang, Heshan Zhou, Xiaoqian Zhang, Zhongxiang Ding, Jianfeng Xu

**Affiliations:** ^1^ Department of Radiology, Affiliated Hangzhou First People’s Hospital, Zhejiang University School of Medicine, Hangzhou, China; ^2^ Department of Radiology, Shulan (Hangzhou) Hospital Affiliated to Zhejiang Shuren University, Shulan International Medical College, Hangzhou, China

**Keywords:** hepatocellular carcinoma, cytokeratin 19, magnetic resonance imaging, nomogram, radiomics

## Abstract

**Objectives:**

We aimed to construct and validate a radiomics-based nomogram model derived from gadoxetic acid–enhanced magnetic resonance (MR) images to predict cytokeratin (CK) 19–positive (+) hepatocellular carcinoma (HCC) and patients’ prognosis.

**Methods:**

A two-center and time-independent cohort of 311 patients were retrospectively enrolled (training cohort, n = 168; internal validation cohort, n = 72; external validation cohort, n = 71). A total of 2286 radiomic features were extracted from multisequence MR images with the uAI Research Portal (uRP), and a radiomic feature model was established. A combined model was established by incorporating the clinic-radiological features and the fusion radiomics signature using logistic regression analysis. Receiver operating characteristic curve (ROC) was used to evaluate the predictive efficacy of these models. Kaplan–Meier survival analysis was used to assess 1-year and 2-year progression-free survival (PFS) and overall survival (OS) in the cohort.

**Results:**

By combining radiomic features extracted in DWI phase, arterial phase, venous and delay phase, the fusion radiomics signature achieved AUCs of 0.865, 0.824, and 0.781 in the training, internal, and external validation cohorts. The final combined clinic-radiological model showed higher AUC values in the three datasets compared with the fusion radiomics model. The nomogram based on the combined model showed satisfactory prediction performance in the training (C-index, 0.914), internal (C-index, 0.855), and external validation (C-index, 0.795) cohort. The 1-year and 2-year PFS and OS of the patients in the CK19+ group were 76% and 73%, and 78% and 68%, respectively. The 1-year and 2-year PFS and OS of the patients in the CK19-negative (−) group were 81% and 77%, and 80% and 74%, respectively. Kaplan–Meier survival analysis showed no significant differences in 1-year PFS and OS between the groups (*P* = 0.273 and 0.290), but it did show differences in 2-year PFS and OS between the groups (*P* = 0.032 and 0.040). Both PFS and OS were lower in CK19+ patients.

**Conclusion:**

The combined model based on clinic-radiological radiomics features can be used for predicting CK19+ HCC noninvasively to assist in the development of personalized treatment.

## Introduction

Hepatocellular carcinoma(HCC) is the sixth most common malignant tumor worldwide in terms of incidence, and it ranks third in terms of mortality rate (1, 2), accounting for approximately 90% of primary liver cancer (3). In China, HCC is the second leading cause of cancer-related deaths (4). The development of HCC is a multistage and multifactorial process, and HCC is heterogeneous and characterized by high tumor invasiveness, frequent recurrence, and poor prognosis (5). Some serum biomarkers were used in the early diagnosis and prognosis of HCC (6, 7). Currently, there is no method that can provide complete information on the diagnosis, prognosis, and treatment outcome of patients with HCC (8).

Cytokeratin 19 (CK19), also known as keratin 19 (KRT19), is a type I acidic 40 kDa keratin encoded by the *KRT19* gene at 17q12-q21. CK19 is a progenitor cell marker or cholangiocyte marker, and approximately 10%–30% of HCCs express CK19 (9). Studies had shown that the expression of CK19 can enhance the proliferation and invasiveness of cells and increase the malignancy of tumors, which was a factor in the poor prognosis of patients (10–12). Wu et al. (13), Qin et al. (14), and Evangelia et al. (15) reported that CK19-positive (+) HCC had a significantly higher incidence of microvascular invasion than CK19-negative (−) HCC, with higher invasiveness and lower disease-free survival. CK19+ HCC had been identified as a new high-risk subtype characterized by strong invasiveness, early recurrence, and poor postoperative prognosis, and it requires more effective treatment.

The preoperative diagnosis of CK19+ HCC mainly relies on pathological examination, which is invasive and inconvenient, which may cause some complications. Some studies (16–18) have predicted CK19 status preoperatively by quantitative or functional magnetic resonance imaging (MRI). In recent years, with the rapid development of artificial intelligence, radiomics has widely been used in the study of tumor classification, prognosis and treatment evaluation (19–24). Radiomics can integrate multiple biomarkers to construct valuable predictive and validation models to guide clinical decision-making. A few studies used radiomics to construct models for predicting CK19 status (25–28). However, some studies had small samples, were based on single-center data, or did not include enhanced sequences or a single sequence, which limited the generalization of the models.

In this study, we aimed to establish a multiparameter radiomics nomogram model as a noninvasive method to predict CK19 status in HCC, providing a basis for clinical individualized treatment. Furthermore, we aimed to investigate the prognostic effect of CK19 on 1-year and 2-year progression-free survival (PFS) and overall survival (OS) in HCC.

## Materials and methods

### Patient population

**Figure 1:**
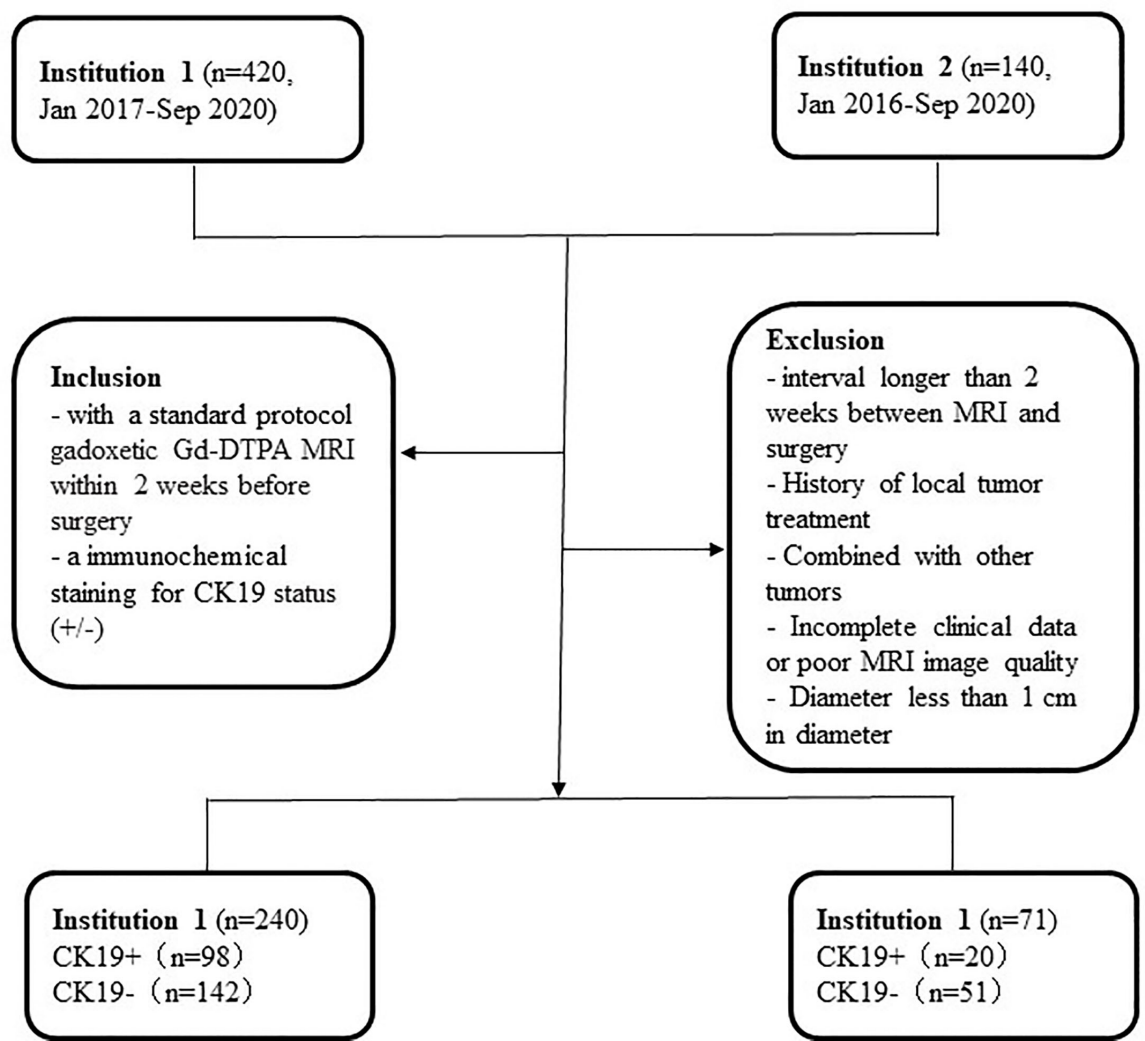
Flowchart of the present study.

This retrospective study was approved by the Medical Ethics Committees of Institutions I and II, and the requirement for informed consent was waived. Data were collected for patients who were confirmed by surgery and pathology from January 2016 to December 2020. The inclusion criteria were as follows: 1) standard-protocol Gadopentetate dimeglumine (Gd-DTPA) MRI conducted within two weeks before surgery; and 2) immunohistochemical staining for CK19 status (+/−) (CK19+ was defined as ≥ 5% of tumor cells staining). The exclusion criteria were as follows: 1) interval longer than two weeks between MRI and surgery; 2) history of local tumor treatment; 3) simultaneous presence of other tumors; 4) incomplete clinical data or poor MRI image quality; and 5) lesion diameter of < 1 cm. The workflow of the study is summarized in **Figure 1**.

### MRI protocols

#### MRI examination methods

End-expiratory hold or respiratory gating method was used for the MRI exam, and the patients did not drink any water for 4h before MRI scanning. Gd-DTPA (Magnevist, Bayer Schering Pharma, Berlin, Germany) was used as the contrast agent. The dose of Gd-DTPA was 0.025 mmol/kg or 0.1 mmol/kg, and the injection velocity was 2 mL/s. Arterial phase (AP), venous phase (VP), and delayed phase (DP) were scanned at 18-23 s, 50-60 s, and 150-180 s after intravenous injection, respectively.

#### Parameters for MRI scanning

GE Signa HDxt 1.5-T MR apparatus (GE, Medical System, Milwaukee, USA) and Siemens Magnetom Skyra 3.0-T MR (Siemens, Healthineers, Berlin and Munich, Germany) with an abdominal 8-channel phased-array coil were used for scanning in Institution I. Siemens Magnetom Verio 3.0-T MR (Siemens, Healthineers, Berlin and Munich, Germany) with abdominal 8-channel phased-array coil was used for scanning in Institution II. Conventional MRI examination sequence was conducted as follows: breath-hold T2-weighted fat-suppressed sequence (T2WI), T1-weighted in-phase and opposed-phase (IP/OP) and free-breath diffusion-weighted imaging (DWI) (b value, 0 and 800 s/mm^2^), T1-weighted fat-suppressed sequence, and three phases of enhancement. Scanning sequences and parameters are shown in **Table 1**.

**Table 1:** Detailed parameters of T1-weighted imaging, T2-weighted imaging, diffusion weighted imaging in each institution.

Scanner	Sequence	TR (ms)	TE (ms)	matrix	FOV (mm^2^)	slice thickness (mm)	Gap (mm)
Institution 1(GE Signa Hdxt 1.5T)	IP/OP	6.1	4.2	224×224	400×400	5	0
	T2WI	4500	90-100	320×192	380×380	6	1.2
	DWI	10588.2	71.5	128×128	380×380	6	1
	T1WI+C	4.2	2.0	320×224	400×400	5	0
Institution 1(SEIMEMS MAGNETOM Skyra 3.0T)	IP/OP	170	1.3	320×256	380×380	5	0
	T2WI	3000	84	320×320	380×380	5	1.2
	DWI	6300	54	126×126	380×380	5	1
	T1WI+C	3.67	1.34	320×240	380×380	3	0
Institution 2(SEIMEMS MAGNETOM Verio 3.0T)	IP/OP	5.49	2.46	320×320	380×380	3	0
	T2WI	5200	80	256×256	380×380	6	1.2
	DWI	10200	70	140×140	380×380	5	1
	T1WI+C	3.92	1.3	256×256	380×380	3	0

DWI, diffusion weighted imaging; FOV, Field of view IP, in-phase; OP, opposed-phase; T1WI, T1-weighted imaging; T2WI, T2-weighted imaging; TE, echo time; TR, repetition time.

#### Morphological features of MRI images

MRI morphological features were assessed by two abdominal radiologists (with 8 years and more than 15 years of experience) by using a picture archiving and communication system (PACS). The following quantitative and qualitative imaging parameters (29) of each HCC were evaluated: (1) tumor size (on the axial plane of diameter); (2) tumor number; (3) tumor margin (smooth or irregular); (4) hemorrhagic component; (5) fat component; (6) arterial rim enhancement; (7) Vp or DP washout (nonperipheral washout or peripheral washout); (8) diffusion restriction; (9) satellite nodule; and (10) macrovascular invasion. The assessors were blind to clinical and pathological data by following an independent assessment. When there was any disagreement, a final decision was made on discordant qualitative parameters in a negotiated manner. Quantitative parameter measurements were averaged. For multiple tumors, the diameter of the largest one was measured.

### Imaging segmentation, feature extraction, and selection

**Figure 2:**
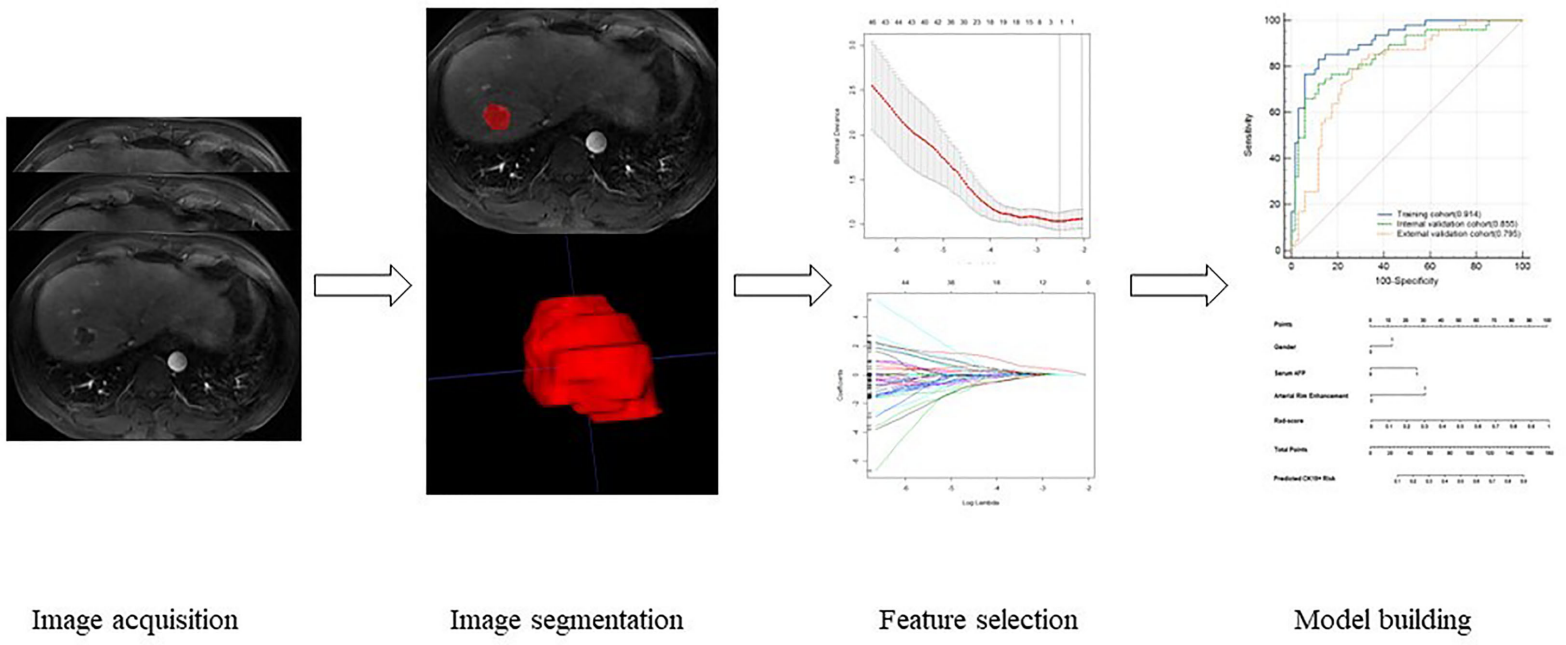
The process of radiomics analysis.

Image segmentation was manually delineated by two radiologists with 8 and more than 15 years of experience in abdominal imaging by uAI Research Portal software (uRP) (United Imaging Intelligence, China) in T1WI, T2WI, DWI, AP, VP, and DP sequences. The regions of interest (ROIs) were delineated layer by layer along the tumor margin, and the delineated ROIs were saved. The delineation schematic is shown in **Figure 2**. After delineation, the radiomics module on the platform was applied for feature extraction, and the features extracted by the two physicians were analyzed for consistency to retain features with intraclass correlation coefficients (ICC) ≥ 0.75. Then, the retained feature data of one of the physicians were normalized using a Z-value normalization algorithm to establish a radiomics model based on radiomic feature parameter score (Rad-score) through minimum absolute tightening and selection operators. Multivariate binary logistic regression analysis were performed for each potential predictor variable, including gender, alpha-fetoprotein(AFP) and morphological features. Finally, least absolute shrinkage and selection operator (LASSO) regression was selected to reduce the dimension of each feature so as to obtain statistically significant radiomics feature parameters, and the Rad-score was also calculated to establish an image-omics model. The process of radiomics analysis was shown in **Figure 2**.

### Model building and external validation

Combined models were constructed by selecting independent risk factors and Rad-score, and then nomograms were made. Following model development, fit was analyzed using the Hosmer–Lemeshow test. ROC curves were plotted, and AUC was used to evaluate the assessment efficacy of each model for CK19+ prediction. Decision curves were used to assess the net benefit of each model.

### Prognostic analysis

All of the patients were followed up as outpatients or by telephone for 1–24 months after surgery or treatment. The presence or absence of tumor progression was identified using enhanced computed tomography (CT) or MRI. Data cutoff was September 30, 2022.

### Statistical analysis

Statistical analysis was conducted by SPSS (version 26.0), MedCalc (version 19.1), and R software (version 4.1.2). Variables of a normal distribution were shown as mean ± SD, and independent-samples *t* test was used for comparison between groups; and Mann–Whitney *U* test was used for comparison between groups. Enumeration data were compared between groups using the *χ2* test. A decision curve was drawn to analyze the net benefit. AUCs were compared between the models using the Delong test. ICC was used to evaluate the measurement agreement between the two ROI-delineating physicians, and ICC ≥ 0.75 was considered to be good agreement. The kappa test was used to compare the agreement between the actual results of external validation data and the predicted results of the combined model, with kappa values ≥ 0.75 as good agreement, 0.4 < kappa values < 0.75 as moderate agreement, and kappa values ≤ 0.4 as poor agreement. Disease-specific survival was computed from the date of surgery to date of death or censored at the date of the last follow-up. Kaplan–Meier survival analysis was used to assess 1-year and 2-year PFS and OS. The Log-rank test was performed to compare survival differences between the two groups. *P* < 0.05 was considered to be statistically significant.

## Results

### Clinical characteristics and conventional MRI findings

A total of 240 patients were randomly divided into the training group (n = 168) and the internal validation group (n = 72) according to the ratio of 7:3. All of the patients were divided into the CK19− group and the CK19+ group according to CK19 staining (Institution I: CK19− group, n = 142; CK19+ group, n = 98; and Institution II: CK19− group, n = 50; CK19+ group, n = 21). The clinical characteristics of the patients in the two groups are compared in **Table 2**. Gender, serum AFP and PIVKA-II were significantly different (*P* < 0.05). There was a higher proportion of females in the CK19+ group.

**Table 2:** Comparison of clinical characteristics according to CK19 status in institution I and institution II.

Characteristics	Institution I (n=240)			Institution II (n=71)		
	CK19+ (n=98)	CK19- (n=142)	*P* value	CK19+ (n=21)	CK19- (n=50)	*P* value
Median age, years (range)	51 (24-75)	53 (27-80)	0.548	55 (30-81)	52 (29-79)	0.499
Gender			0.012			0.047
Male	68 (69%)	118 (83%)		12 (57%)	40 (80%)	
Female	30 (31%)	24 (17%)		9 (43%)	10 (20%)	
HBV			0.212			0.986
Yes	78 (80%)	103 (73%)		16 (76%)	38 (76%)	
No	20 (20%)	39 (27%)		5 (24%)	12 (24%)	
Serum AFP			0.013			0.020
<400ng/ml	53 (54%)	99 (70%)		10 (48%)	38 (76%)	
≥400 ng/ml	45 (46%)	43 (30%)		11 (52%)	12 (24%)	
PIVKA-II			0.041			0.049
<20μg/L	16 (16%)	41 (29%)		3 (14%)	18 (36%)	
≥20μg/L	50 (51%)	63 (44%)		13 (62%)	20 (40%)	
N/A^*^	32 (33%)	38 (27%)		5 (24%)	12 (24%)	
Child-Pugh class			0.402			0.620
A	64 (65%)	100 (70%)		13 (62%)	34 (68%)	
B	34 (35%)	42 (30%)		8 (38%)	16 (32%)	
C	0 (0)	0 (0)		0 (0)	0 (0)	
BCLC stage			0.397			0.706
0/A	30 (31%)	51 (36%)		8 (38%)	16 (32%)	
B	43 (44%)	50 (35%)		7 (33%)	22 (44%)	
C	25 (25%)	41 (29%)		6 (29%)	12 (24%)	
D	0 (0)	0 (0)		0 (0)	0 (0)	

*No data. AFP, Alpha-fetoprotein; BCLC, Barcelona Clinic Liver Cancer; CK19, Cytokeratin 19; HBV, Hepatitis B virus. P< 0.05.

MRI features included tumor size, tumor number, tumor margin, hemorrhagic component, fat component, arterial rim enhancement, VP or DP washout, diffusion restriction, satellite nodule, and macrovascular invasion. Arterial rim enhancement was significantly different in both institutions I and II (*P* < 0.05). However, other features were not significantly different between the two groups (*P* > 0.05). The detailed distribution of MRI features in the two groups is summarized in **Table 3**.

**Table 3:** MRI semantic features of institution I and institution II.

Morohologic features	Institution I (n=240)			Institution II (n=71)		
	CK19+ (n=98)	CK19- (n=142)	P value	CK19+ (n=21)	CK19- (n=50)	P value
Median Tumor size (range,cm)	5.1 ± 3.5 (1.1-14.9)	5.0 ± 3.3 (1.1-13.7)	0.602	5.5 ± 3.0 (1.9-12.3)	5.2 ± 3.0 (1.6-13.6)	0.486
Tumor size			0.590			0.243
≥5cm	45 (46%)	61 (43%)		12 (57%)	21 (42%)	
<5cm	53 (54%)	81 (57%)		9 (43%)	29 (58%)	
Tumor number			0.635			0.782
single	63 (64%)	87 (61%)		14 (67%)	35 (70%)	
multiple	35 (36%)	55 (39%)		7 (33%)	15 (30%)	
Tumor margin			0.377			0.200
smooth	63 (64%)	99 (70%)		10 (48%)	32 (64%)	
irregular	35 (36%)	43 (30%)		11 (52%)	18 (36%)	
Hemorrhagic component			0.797			0.742
Yes	34 (35%)	47 (33%)		8 (38%)	17 (34%)	
No	64 (65%)	95 (67%)		13 (62%)	33 (66%)	
Fat component			0.929			0.485
Yes	10 (10%)	15 (11%)		4 (19%)	8 (16%)	
No	88 (90%)	127 (89%)		17 (81%)	42 (84%)	
Arterial rim enhancement			0.001			0.034
Yes	30 (31%)	18 (13%)		7 (33%)	6 (12%)	
No	68 (69%)	124 (87%)		14 (67%)	44 (88%)	
VP or DP washout						
Nonperipheral washout	80 (82%)	118 (83%)	0.769	18 (86%)	44 (88%)	0.792
Peripheral washout	18 (18%)	24 (17%)		3 (14%)	6 (12%)	
Diffusion restriction			0.859			0.859
Yes	87 (89%)	125 (88%)		17 (81%)	43 (86%)	
No	11 (11%)	17 (12%)		4 (19%)	7 (14%)	
Satellite nodule			0.635			0.782
Yes	63 (64%)	87 (61%)		14 (67%)	35 (70%)	
No	35 (36%)	55 (39%)		7 (33%)	15 (30%)	
Macrovasular invasion			0.566			0.686
Yes	25 (26%)	41 (29%)		6 (29%)	12 (24%)	
No	73 (74%)	101 (71%)		15 (71%)	38 (76%)	

CK19, cytokeratin 19; DP, delayed phase; VP, portal venous phase.

According to univariate and multivariate logistic regression analysis, gender (OR = 7.233, *P* < 0.05), AFP (OR = 3.167, *P* < 0.05), and arterial rim enhancement (OR = 4.656, *P* < 0.05) were independent variables associated with CK19 status (**Table 4**). The clinical model with these factors had AUCs of 0.749, 0.740 and 0.778 in the training cohort, internal and external validation cohort respectively.

**Table 4:** Univariate and Multivariate analysis logistic regression of variables in predicting CK19 status.

Factors	Univariate		Multivariate	
	OR (95%*CI*)	*P* value	OR (95%*CI*)	*P* value
Age (≥65/<65 years)	1.753 (0.976-4.114)	0.351		
Gender (Female/Male)	8.418 (1.301-16.112)	0.009	7.233 (1.434-15.255)	0.042
HBV (+/-)	2.877 (1.368-5.261)	0.277		
Serum AFP (≥400/<400 ng/ml)	3.702 (1.561-6.887)	< 0.001	3.167 (1.452-6.716)	0.018
PIVKA-II (≥20/<20μg/L)	1.540 (0.634-3.256)	0.019	0.980 (0.212-2.098)	0.206
Child-Pugh class (B/A)	1.427 (0.799-2.379)	0.369		
BCLC stage				
0/A	1.482 (0.885-4.018)	0.109		
B	1.510 (1.107-3.231)	0.315		
C	1.346 (0.697-3.052)	0.238		
Tumor size (≥5/<5 cm)	1.305 (0.689-2.817)	0.712		
Tumor number (≥2/<2)	1.561 (1.166-4.460)	0.295		
Tumor margin (irregular/smooth)	2.280 (1.503-4.849)	0.534		
Hemorrhagic component (yes/no)	1.664 (1.252-5.770)	0.156		
Fat component (yes/no)	2.046 (1.004-4.907)	0.580		
Arterial rim enhancement (yes/no)	4.973 (2.192-9.841)	< 0.001	4.656 (1.679-10.381)	0.013
VP or DP washout (yes/no)	0.948 (0.529-1.945)	0.727		
Diffusion restriction (yes/no)	1.234 (0.613-2.679)	0.114		
Satellite nodule (yes/no)	3.251 (2.438-5.825)	0.330		
Macrovasular invasion (yes/no)	5.461 (3.358-8.490)	0.601		

AFP, Alpha-fetoprotein; BCLC, Barcelona Clinic Liver Cancer; CK19, Cytokeratin 19; DP, Delayed Phase; HBV, Hepatitis B virus. VP, Portal Venous Phase. P< 0.05.

### Consistency analysis

The ICC range by two radiologists was 0.551–0.936, and 2090 out of 2286 (91.4%) radiomics features and all of the semantic features had good consistency (ICC ≥ 0.75). Therefore, the data of senior doctors were used for feature selection and modeling.

### Radiomics analysis

The radiomics features of six sequences were selected through uRP. Finally, 0, 3, 6, 8, 7, and 10 features were selected in T1WI, T2WI, DWI, AP, VP, and DP sequences, respectively. A total of 34 features were further selected with binary logistic regression, and 6 features were finally selected in the fusion radiomics model, namely, DWI-log_glcm_log-sigma-0-5-mm-3D-JointEn-tropy (LGLS), AP [recursivegaussian_ gldm_ LargeDependenceHighGrayLevelEmphasis (RGL), wavelet_glszm_wavelet- LLL- HighGrayLevelZoneEmphasis (WGWLH), curvatureflow_gldm_DependenceNonUniformity (CGD)], VP wavelet_glcm_wavelet-LHH-Idnd2 (WG-WLI), and DPGLCMEnergy_angle135_offset7. According to the weighted sum of the correlation coefficients, the Rad-score was obtained.

Rad-score=-0.00583932*DWI-log_glcm_log-sigma-0-5-mm-3D-JointEn-tropy (LGLS)+ 0.091226846*AP-recursivegaussian_ gldm_ LargeDependenceHighGrayLevelEmphasis (RGL)+ 0.014082735*AP-wavelet_glszm_wavelet- LLL- HighGrayLevelZoneEmphasis (WGWLH)+ 0.005515757*AP-curvatureflow_gldm_DependenceNonUniformity (CGD)+ 0.014082735*VP-wavelet_glcm_wavelet-LHH-Idnd2 (WG-WLI)+ -0.073718965*DP-GLCMEnergy_angle135_offset7.

**Figure 3:**
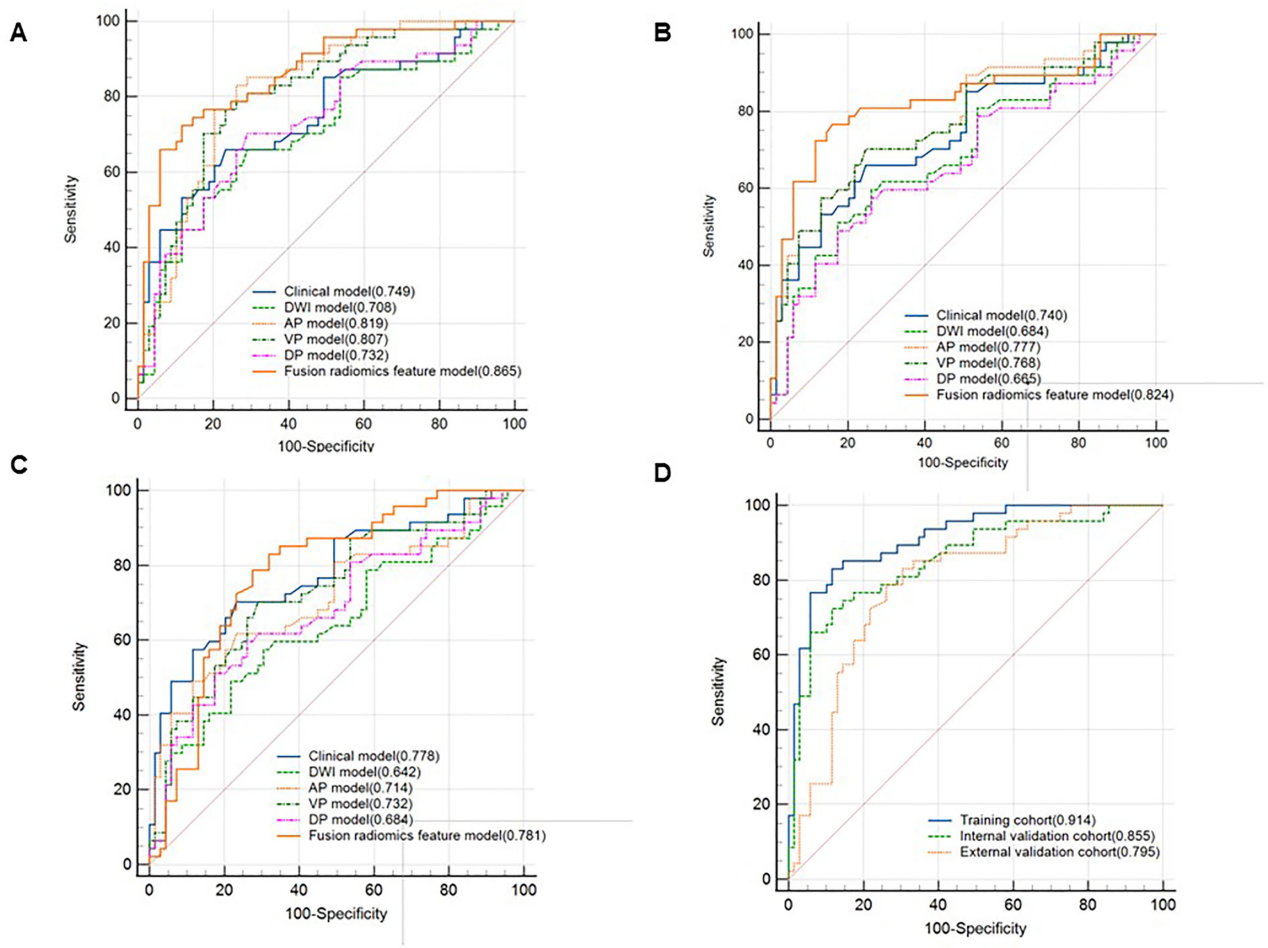
Comparison of receiver operating characteristics (ROC) curves for predicting CK19 status. ROC curves of the clinical, DWI, AP, VP, DP and fusion radiomics feature model in the training cohort **(A)**, internal validation cohort **(B)** and external validation cohort **(C)**. ROC curves of combined clinical with fusion radiomics feature model in the three cohorts **(D)**.

In the training cohort, the AUCs ranged from 0.708 to 0.819 for DWI, AP, VP, and DP sequences, where AP showed the best performance with an AUC of 0.819 (95% CI [0.737, 0.884], *P* < 0.001) (**Figure 3**). Fusion radiomics feature models achieved AUCs of 0.865, 0.824, and 0.781 in the training, internal, and external validation datasets, respectively **(** **Figure 3**, **Table 5** **).** The final combined clinic-radiological model showed higher AUC values in the three datasets than the fusion radiomics model **(** **Table 5** **)**.

**Table 5:** Predictive efficacy of different models.

Models	Training cohort(n=168)				Internal validation cohort (n=72)				External validation cohort (n=71)			
	AUC	Sensitivity(%)	Specificity(%)	95%*CI*	AUC	Sensitivity(%)	Specificity(%)	95%*CI*	AUC	Sensitivity(%)	Specificity(%)	95%*CI*
Clinical model	0.749	68.5	77.8	0.660-0.825	0.740	67.8	70.0	0.650-0.817	0.778	72.6	69.9	0.691-0.849
DWI model	0.708	68.7	76.1	0.616-0.789	0.684	69.8	77.7	0.591-0.767	0.642	90.1	62.1	0.548-0.729
AP model	0.819	76.1	88.3	0.737-0.884	0.777	80.0	69.5	0.691-0.849	0.714	58.7	78.9	0.623-0.794
VP model	0.807	80.7	76.5	0.723-0.874	0.768	78.7	81.4	0.680-0.841	0.732	67.5	83.4	0.642-0.810
DP model	0.732	70.4.3	78.4	0.642-0.810	0.665	83.9	70.0	0.571-0.750	0.684	80.0	71.0	0.591-0.767
Fusion radiomics feature model	0.867	85.6	81.0	0.789-0.921	0.824	80.1	79.4	0.745-0.890	0.781	86.3	75.2	0.695-0.853
Combined model	0.914	81.8	96.5	0.848-0.958	0.855	78.9	81.8	0.777-0.913	0.795	76.9	81.8	0.711-0.865

### Nomogram building and validation

**Figure 4:**
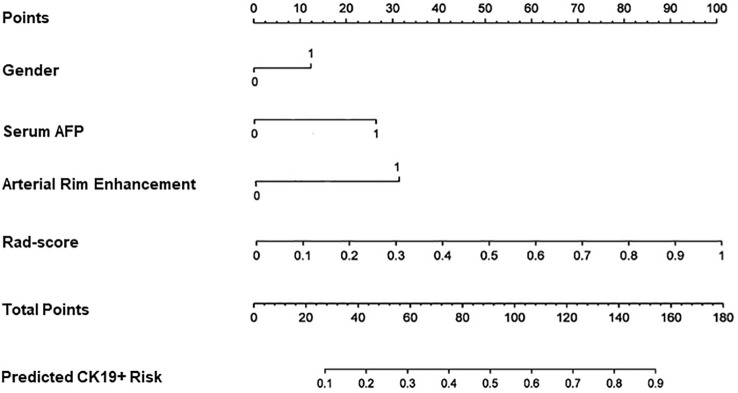
Nomogram for predicting the positive of CK19. Gender, serum AFP and arterial rim enhancement were the risk factors. For gender “0” refers to male, ”1” refers to female. For arterial rim enhancement, “0” refers to absence of the feature and “1” refers to presence of it. “Rad-score” is the prediction of CK19 status of the fusion radiomics model. “Total points” is the total score by adding gender, serum AFP, arterial rim enhancement, and rad-score.

Four features, namely, Rad-score, gender, serum AFP, and arterial rim enhancement were used as risk factors for predicting the status of CK19 to establish the combined model and obtain the nomogram **(** **Figure 4** **).** The nomogram based on the combined model showed satisfactory prediction performance in the training (C-index, 0.914), internal (C-index, 0.855), and external validation (C-index, 0.795) cohorts(**Figure 3D**).

### Prognosis

**Figure 5:**
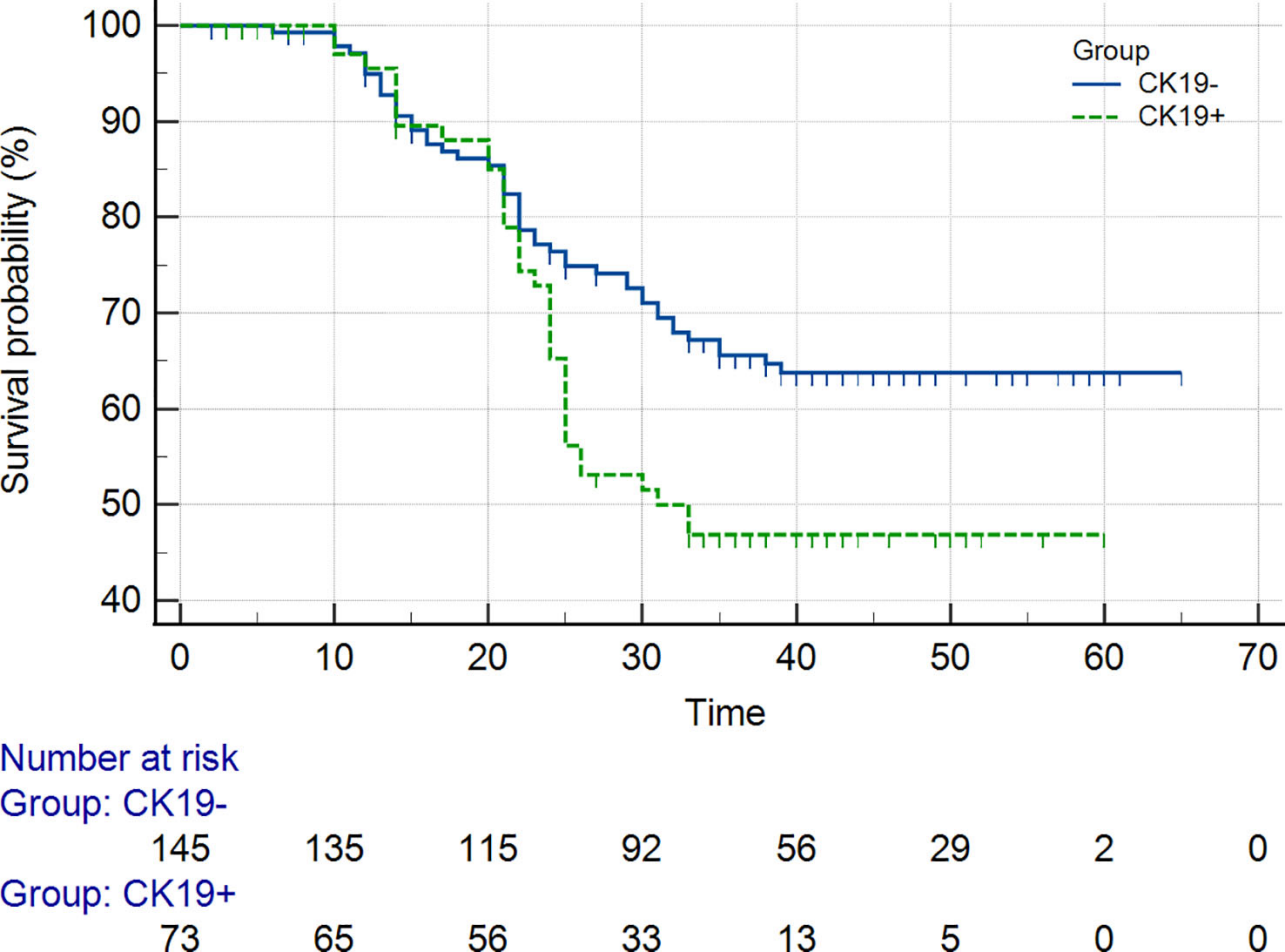
Kaplan-Meier curves showed overall survival (OS).

As of September 2022, 218 out of 311 (70.1%) patients had completed the PFS and OS follow-up, including the CK19+ group (n = 73) and the CK19− group (n = 145) in the two institutions. The overall recurrence rate was 46.8% (102/218), and the overall death rate was 29.8% (65/218). The 1-year and 2-year PFS and 1-year and 2-year OS of the patients in the CK19+ group were 76% and 73%, and 78% and 68%, respectively. The 1-year and 2-year PFS and 1-year and 2-year OS of the patients in the CK19− group were 81% and 77%, and 80% and 74%, respectively. Log-rank Test showed no significant differences in 1-year PFS and OS between the groups (*P* = 0.273 and 0.290), but differences were found in 2-year PFS and OS between the groups (*P* = 0.032 and 0.040). Both PFS and OS were lower in the CK19+ patients **(** **Figure 5** **)**.

## Discussion

In this study, we developed a combined model based on multiple MRI radiomics, clinical features, and qualitative MRI features to predict CK19+ status of HCC. The results showed that the combined model of radiomics labels, AFP ≥ 400 ng/mL, and arterial rim enhancement had good performance and model calibration, which may provide a preoperative evaluation tool to predict CK19 status and guide individualized management in HCC patients.

AFP is the most commonly used tumor marker in clinical and has a degree of specificity for the diagnosis of HCC. Many studies have utilized AFP as a targeted biomarker for treatment or prediction (30, 31). In this study, AFP ≥ 400 ng/mL was found to be an independent risk factor for CK19+ HCC, which was consistent with previous studies (26). Gender was also a risk factor in our cohort, with a higher proportion in CK19+ females, which was not reported in previous studies. It may be associated with oestrogen, but more samples are needed to explore this hypothesis. In our study, arterial rim enhancement was the only valuable feature for multiple MRI semantic feature analysis. Andrea et al. (32) showed ring-enhanced lesions developed faster in early model. Other studies had also shown that arterial rim enhancement suggests that tumors are more aggressive and with a high risk of recurrence (18, 29, 33). Chen et al. (27) showed that intratumoral hemorrhage and peritumor hypointensity on hepatobiliary phase(HBP) were risk factors for CK19+ HCC. There was no significant difference in hemorrhage component in our cohort, which differed from previous studies. We were unable to assess qualitative features of HBP without the use of liver-specific acetic acid. Differences in the assessment of qualitative characteristics across studies may be due to subjective factors and lack of standardization. Numerous studies have identified CK19 as an important factor in poor prognosis after hepatectomy for HCC (10–12, 34). CK19 expression is also closely associated with microvascular invasion (MVI), and HCC with high CK19 expression has more frequent MVI, stronger invasiveness, higher recurrence rates, and lower survival rates. In present study, APF and arterial rim enhancement were high-risk factors for CK19+ HCC, which confirms the high invasiveness of this subtype of HCC.

In our study, a total of seven radiomics constructs combined radiomics features from DWI (n = 1), AP (n = 3), VP (n = 1), and DP (n = 1). The AUCs were 0.865, 0.824, and 0.781 in the training, internal validation, and external validation datasets, respectively. The AUCs of the final combined clinic-radiological model were 0.914, 0.855, and 0.795, which were higher than those in the fusion radiomics model. Sensitivity and specificity were more than 77%. Although the AUC of the training set in our study was lower than that reported by Wang et al. (25), the internal validation results showed better performance, while the external validation also demonstrated good generalization of this model. Multiple nomogram models have been successfully applied in the study of clinical lesions, such as nomogram models predicting HCC patients with microvascular invasion, tumor differentiation, and immunoscore (35, 36). In our study, a nomogram was established based on the clinical, MRI semantic features and radiomics features to predict the preoperatively CK19 status in HCC patients. The enhanced MRI nomogram model was able to obtain the probability of CK19+ status through simple addition operation, which was conducive to personalized prediction of patients. The AUC of the model was 0.795-0.914 and the model had better predictive efficacy.

Previous studies demonstrated poor prognosis and high recurrence in patients with CK 19+ HCC. Lee et al. (37) showed that the OS rate of CK19⁻ HCC and CK 19+ HCC was 90.7% and 74.3%, respectively, at 1-year after the resection. In our study the 1-year OS rate of CK19⁻ HCC and CK19+ HCC was 77% and 73% lower than Lee et al. This difference may be due to difference in the inclusion criteria, and some patients with higher stage who were ineligible for surgery. In the present study, the 1-year and 2-year PFS and OS were worse for CK19+ group than for CK19-negative patients, which was consistent with Wu et al (13). While there were no significant differences in PFS and OS in patients with CK19+/- HCC based on 1-year of follow up, 2-year PFS and OS had significant differences. This suggests that CK19+ HCC is more likely to be highly invasive than CK19- HCC, and that active postoperative management is required.

There were some limitations to our study. First, it was a retrospective study and may have selection bias. Second, liver-specific acetic acid (gadolinium ethoxybenzyl-diethylenetriaminepenta-acetic acid, Gd-EOB-DTPA) was not injected into the patients in this study. The hepatobiliary phase may provide more evidence (14), and comparison with the models was lacking. Third, CK19 is also expressed in bile duct cell carcinoma and dual-phenotype hepatocellular carcinoma; whether our models can accurately differentiate CK19+ HCC from these tumors remains to be further studied. In the future, more large, multicenter, prospective studies are needed to validate radiomic as a noninvasive marker.

## Conclusion

In conclusion, the combined model based on the clinic-radiological radiomics features showed good performance, and it can be used for predicting CK19+ HCC noninvasively. The model can help to develop personalized treatment and predict recurrence and prognosis in HCC more accurately.

## Data availability statement

The raw data supporting the conclusions of this article will be made available by the authors, without undue reservation.

## Ethics statement

This retrospective study was approved by the Medical Ethics Committees of Institutions I and II, and the requirement for informed consent was waived.

## Author contributions

LZ carried out the studies, participated in collecting data, and drafted the manuscript. HZ and XZ contributed to the data acquisition, analysis, and interpretation. JX designed the study. ZD helped to draft the manuscript. All authors contributed to the article and approved the submitted version.

## References

[1] Sung H Ferlay J Siegel RL Laversanne M Soerjomataram I Jemal A . Global cancer statistics 2020: GLOBOCAN estimates of incidence and mortality worldwide for 36 cancers in 185 countries. CA Cancer J Clin (2021) 71:209–49. doi: 10.3322/caac.21660 33538338

[2] Chen X Chi H Zhao X Pan R Wei Y Han Y . Role of exosomes in immune microenvironment of hepatocellular carcinoma. J Oncol (2022) 2022:2521025. doi: 10.1155/2022/2521025 35126514 PMC8816547

[3] Galle PR Forner A JM L Mazzaferro V Piscaglia F Raoul J-L . EASL clinical practice guidelines: management of hepatocellular carcinoma. J Hepatol (2018) 69:182–236. doi: 10.1016/j.jhep.2018.03.019 29628281

[4] Xia C Dong X Li H Cao M Sun D He S . Cancer statistics in China and united states, 2022: profiles, trends, and determinants. Chin Med J (Engl) (2022) 135:584–90. doi: 10.1097/CM9.0000000000002108 PMC8920425 35143424

[5] Li H Wu Z Chen J Su K Guo L Xu K . External radiotherapy combined with sorafenib has better efficacy in unresectable hepatocellular carcinoma: a systematic review and meta-analysis. Clin Exp Med (2022). doi: 10.1007/s10238-022-00972-4 PMC10460724 36495367

[6] Su K Liu Y Wang P He K Wang F Chi H . Heat-shock protein 90α is a potential prognostic and predictive biomarker in hepatocellular carcinoma: a large-scale and multicenter study. Hepatol Int (2022) 16:1208–19. doi: 10.1007/s12072-022-10391-y PMC9525341 35972640

[7] Feng H Li B Li Z Wei Q Ren L . PIVKA-II serves as a potential biomarker that complements AFP for the diagnosis of hepatocellular carcinoma. BMC Cancer (2021) 21:401. doi: 10.1186/s12885-021-08138-3 33849479 PMC8045263

[8] Shuai H Fan M Hongkai Z Hailiang L Jinrong Q . Correlation analysis of Ki67, Ck19 with clinicopathological features and apparent diffusion coefficient value of hepatocellular carcinoma. Natl Med J China (2021) 101:798–802. doi: 10.3760/cma.j.cn112137-20210108-00058 33765721

[9] Zhuo J-Y Lu D Tan W-Y Zheng S-S Shen Y-Q Xu X . CK19-positive hepatocellular carcinoma is a characteristic subtype. J Cancer (2020) 11:5069–77. doi: 10.7150/jca.44697 PMC7378918 32742454

[10] Rhee H Kim H Park YN . Clinico-Radio-Pathological and molecular features of hepatocellular carcinomas with keratin 19 expression. Liver Cancer (2020) 9:663–81. doi: 10.1159/000510522 PMC7768132 33442539

[11] Shuyao W Mingyang B Feifei M Xiaoqin H . CK19 predicts recurrence and prognosis of HBV positive HCC. J Gastrointest Surg Off J Soc Surg Aliment Tract (2022) 26:341–51. doi: 10.1007/s11605-021-05107-w 34506016

[12] Sun D Zhang Y Sun X Chen Y Qiu W Ji M . Prognostic value of cytokeratin 19 in hepatocellular carcinoma: a meta-analysis. Clin Chim Acta Int J Clin Chem (2015) 448:161–9. doi: 10.1016/j.cca.2015.06.027 26164382

[13] Wu M-S Zhong J-H Chen K Luo C-P Zhang J Zhou Y-J . Association of CK19 expression with the efficacy of adjuvant transarterial chemoembolization after hepatic resection in hepatocellular carcinoma patients at high risk of recurrence. J Clin Transl Res (2022) 8:71–9. PMC8872984 35224238

[14] Qin S-D Zhang J Qi Y-P Zhong J-H Xiang B-D . Individual and joint influence of cytokeratin 19 and microvascular invasion on the prognosis of patients with hepatocellular carcinoma after hepatectomy. World J Surg Oncol (2022) 20:209. doi: 10.1186/s12957-022-02632-z 35725470 PMC9210815

[15] Fatourou E Koskinas J Karandrea D Palaiologou M Syminelaki T Karanikolas M . Keratin 19 protein expression is an independent predictor of survival in human hepatocellular carcinoma. Eur J Gastroenterol Hepatol (2015) 27:1094–102. doi: 10.1097/MEG.0000000000000398 26011233

[16] Guo Y Chen J Zhang Y Guo Y Jiang M Dai Y . Differentiating cytokeratin 19 expression of hepatocellular carcinoma by using multi-b-value diffusion-weighted MR imaging with mono-exponential, stretched exponential, intravoxel incoherent motion, diffusion kurtosis imaging and fractional order calculus models. Eur J Radiol (2022) 150:110237. doi: 10.1016/j.ejrad.2022.110237 35278979

[17] Chen J Liu D Guo Y Zhang Y Guo Y Jiang M . Preoperative identification of cytokeratin 19 status of hepatocellular carcinoma based on diffusion kurtosis imaging. Abdom Radiol N Y (2023) 48:579–89. doi: 10.1007/s00261-022-03736-6 36416905

[18] Choi S-Y Kim SH Park CK Min JH Lee JE Choi Y-H . Imaging features of gadoxetic acid–enhanced and diffusion-weighted MR imaging for identifying cytokeratin 19-positive hepatocellular carcinoma: a retrospective observational study. Radiology (2018) 286:897–908. doi: 10.1148/radiol.2017162846 29166246

[19] Ji G-W Zhu F-P Xu Q Wang K Wu M-Y Tang W-W . Radiomic features at contrast-enhanced CT predict recurrence in early stage hepatocellular carcinoma: a multi-institutional study. Radiology (2020) 294:568–79. doi: 10.1148/radiol.2020191470 31934830

[20] Kong C Zhao Z Chen W Lv X Shu G Ye M . Prediction of tumor response *via* a pretreatment MRI radiomics-based nomogram in HCC treated with TACE. Eur Radiol (2021) 31:7500–11. doi: 10.1007/s00330-021-07910-0 PMC8452577 33860832

[21] Wu J-P Ding W-Z Wang Y-L Liu S Zhang X-Q Yang Q . Radiomics analysis of ultrasound to predict recurrence of hepatocellular carcinoma after microwave ablation. Int J Hyperth Off J Eur Soc Hyperthermic Oncol North Am Hyperth Group (2022) 39:595–604. doi: 10.1080/02656736.2022.2062463 35435082

[22] He Y Hu B Zhu C Xu W Ge Y Hao X . A novel multimodal radiomics model for predicting prognosis of resected hepatocellular carcinoma. Front Oncol (2022) 12:745258. doi: 10.3389/fonc.2022.745258 35321432 PMC8936674

[23] Luo J Huang Z Wang M Li T Huang J . Prognostic role of multiparameter MRI and radiomics in progression of advanced unresectable hepatocellular carcinoma following combined transcatheter arterial chemoembolization and lenvatinib therapy. BMC Gastroenterol (2022) 22:108. doi: 10.1186/s12876-022-02129-9 35260095 PMC8903551

[24] Liu Q-P Yang K-L Xu X Liu X-S Qu J-R Zhang Y-D . Radiomics analysis of pretreatment MRI in predicting tumor response and outcome in hepatocellular carcinoma with transarterial chemoembolization: a two-center collaborative study. Abdom Radiol N Y (2022) 47:651–63. doi: 10.1007/s00261-021-03375-3 34918174

[25] Wang W Gu D Wei J Ding Y Yang L Zhu K . A radiomics-based biomarker for cytokeratin 19 status of hepatocellular carcinoma with gadoxetic acid–enhanced MRI. Eur Radiol (2020) 30:3004–14. doi: 10.1007/s00330-019-06585-y 32002645

[26] Wang H Yang C Zeng M Rao S Ji Y Weng X . Magnetic resonance texture analysis for the identification of cytokeratin 19-positive hepatocellular carcinoma. Eur J Radiol (2019) 117:164–70. doi: 10.1016/j.ejrad.2019.06.016 31307643

[27] Chen Y Chen J Zhang Y Lin Z Wang M Huang L . Preoperative prediction of cytokeratin 19 expression for hepatocellular carcinoma with deep learning radiomics based on gadoxetic acid-enhanced magnetic resonance imaging. J Hepatocell Carcinoma (2021) 8:795–808. doi: 10.2147/JHC.S313879 34327180 PMC8314931

[28] Yang F Wan Y Xu L Wu Y Shen X Wang J . MRI-Radiomics prediction for cytokeratin 19-positive hepatocellular carcinoma: a multicenter study. Front Oncol (2021) 11:672126. doi: 10.3389/fonc.2021.672126 34476208 PMC8406635

[29] Kang H-J Kim H Lee DH Hur BY Hwang YJ Suh K-S . Gadoxetate-enhanced MRI features of proliferative hepatocellular carcinoma are prognostic after surgery. Radiology (2021) 300:572–82. doi: 10.1148/radiol.2021204352 34227881

[30] Galle PR Foerster F Kudo M Chan SL Llovet JM Qin S . Biology and significance of alpha-fetoprotein in hepatocellular carcinoma. Liver Int Off J Int Assoc Study Liver (2019) 39:2214–29. doi: 10.1111/liv.14223 31436873

[31] Johnson P Zhou Q Dao DY Lo YMD . Circulating biomarkers in the diagnosis and management of hepatocellular carcinoma. Nat Rev Gastroenterol Hepatol (2022) 19:670–81. doi: 10.1038/s41575-022-00620-y 35676420

[32] Kierans AS Leonardou P Hayashi P Brubaker LM Elazzazi M Shaikh F . MRI Findings of rapidly progressive hepatocellular carcinoma. Magn Reson Imaging (2010) 28:790–6. doi: 10.1016/j.mri.2010.03.005 20427139

[33] An C Kim DW Park Y-N Chung YE Rhee H Kim M-J . Single hepatocellular carcinoma: preoperative MR imaging to predict early recurrence after curative resection. Radiology (2015) 276:433–43. doi: 10.1148/radiol.15142394 25751229

[34] Jin Y Liang Z-Y Zhou W-X Zhou L . Combination with CK19 might increase the prognostic power of hep par 1 in hepatocellular carcinoma after curative resection. J Investig Surg Off J Acad Surg Res (2018) 31:412–9. doi: 10.1080/08941939.2017.1347218 28758812

[35] Tang M Zhou Q Huang M Sun K Wu T Li X . Nomogram development and validation to predict hepatocellular carcinoma tumor behavior by preoperative gadoxetic acid-enhanced MRI. Eur Radiol (2021) 31:8615–27. doi: 10.1007/s00330-021-07941-7 33877387

[36] Yang L Gu D Wei J Yang C Rao S Wang W . A radiomics nomogram for preoperative prediction of microvascular invasion in hepatocellular carcinoma. Liver Cancer (2019) 8:373–86. doi: 10.1159/000494099 PMC6873064 31768346

[37] Lee JI Lee J-W Kim JM Kim JK Chung HJ Kim YS . Prognosis of hepatocellular carcinoma expressing cytokeratin 19: comparison with other liver cancers. World J Gastroenterol (2012) 18:4751–7. doi: 10.3748/wjg.v18.i34.4751 PMC3442214 23002345

